# Supplementary figures and images for: Seroprevalence and risk factors for hepatitis B and hepatitis C in three large regions of Kazakhstan
Source: PLoS One. 2021 Dec 16;16(12):e0261155. doi: 10.1371/journal.pone.0261155 (PMC8675652; doi:10.1371/journal.pone.0261155)

**S1 Appendix. Flowchart of inclusion and exclusion of participants.**

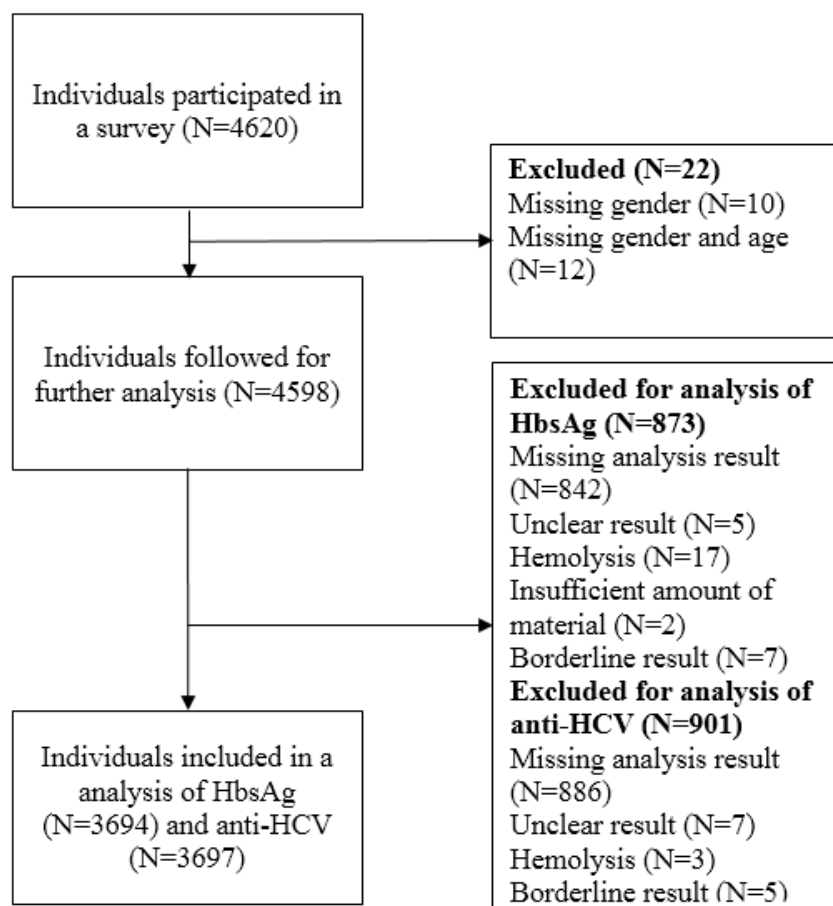

Supplement: S1 Appendix — (PDF) [file pone.0261155.s001.pdf]
