# Supplementary material for: Seroprevalence and risk factors for hepatitis B and hepatitis C in three large regions of Kazakhstan
Source: PLoS One. 2021 Dec 16;16(12):e0261155. doi: 10.1371/journal.pone.0261155 (PMC8675652; doi:10.1371/journal.pone.0261155)
Supplement: S2 Appendix — (PDF) [file pone.0261155.s002.pdf]

S2 Appendix. Global Moran's I test for spatial autocorrelation of HBsAg and anti-HCV seropositivity by region.

Regions	HBsAg seropositivity			Anti-HCV seropositivity		
	Moran's I	Z-score	p-value	Moran's I	Z-score	p-value
All sample	0.006	7.77	<0.001	0.005	6.64	<0.001
West Kazakhstan	-0.001	-0.15	1.12	0.001	2.02	0.04
South Kazakhstan	-0.001	0.32	0.75	-0.001	0.14	0.89
North Kazakhstan	0.001	1.85	0.06	-0.001	-0.60	1.45
