# Supplementary material for: Seroprevalence and risk factors for hepatitis B and hepatitis C in three large regions of Kazakhstan
Source: PLoS One. 2021 Dec 16;16(12):e0261155. doi: 10.1371/journal.pone.0261155 (PMC8675652; doi:10.1371/journal.pone.0261155)
Supplement: S3 Appendix — (PDF) [file pone.0261155.s003.pdf]

S3 Appendix. Russian translated the WHO STEPS instrument.

Анкета по эпиднадзору
факторов риска хронических заболеваний
Казахстан/
Алматы и Алматинская область, Актобе и Актюбинская область

Общая информация

Место и дата		Ответ	Код
1	Код центра/населенного пункта		I1
2	Название центра/населенного пункта	└─┴─┴─┘	I2
3	Идентификационный номер опрашивающего	└─┴─┴─┘	I3
4	Дата заполнения вопросника	└─┴─┘ └─┴─┘ └─┴─┴─┴─┘<br>день месяц год	I4

Идентификационный номер участника └─┴─┴─┴─┴─┴─┘			
**Получение согласия, язык проведения опроса и имя**		**Ответ**	**Код**
5	Согласие участника получено (устно или письменно)	Да 1<br>Нет 2 **Если НЕТ, ПРЕКРАТИТЕ ОПРОС**	I5
6	Язык проведения опроса [укажите]	Казахский 1<br>Русский 2	I6
7	Время проведения опроса (по 24-часовой шкале)	└─┴─┘ : └─┴─┘<br>часы минуты	I7
8	Фамилия		I8
9	Имя		I9
**Дополнительная информация, которая может быть полезной**			
10	Номер телефона для контактов, если возможно		I10

Запишите и храните идентификационную информацию (I5 - I10) отдельно от заполненного вопросника.

Этап 1 Демографическая информация

ОСНОВНОЙ МОДУЛЬ: Демографическая информация				
**Вопросы**		**Ответы**		**Код**
11	Пол (мужской / *женский, по внешним признакам)*	Мужской<br>Женский	1<br>2	C1
12	Когда Вы родились?<br>*Не знаю 77 777 7777*	└─┴─┘ └─┴─┘ └─┴─┴─┴─┘ *Если известно,*<br>*Перейдите к C4*<br>день месяц год		C2
13	Сколько Вам лет?	Годы	└─┴─┘	C3
14	Сколько всего лет Вы учились в режиме полного дня в школе или другом учебном заведении (исключая дошкольное обучение)?	Годы	└─┴─┘	C4

РАСШИРЕННЫЙ МОДУЛЬ: Демографическая информация				
**Вопросы**		**Ответы**		**Код**
16	К какой национальности Вы относитесь?	Казах<br>Русский<br>Узбек<br>Украинец<br>Другая азиатская<br>Другая европейская<br>Отказывается отвечать	1<br>2<br>3<br>4<br>5<br>6<br>88	C6
15	Каков наивысший уровень полученного Вами образования?	Нет школьного образования<br>Начальное образование<br>Неполное среднее (8-9 классов)<br>Среднее (10-11 классов)<br>Среднее профессиональное (колледж, техникум, профессиональное училище)<br>Неоконченное высшее<br>Высшее<br>Отказывается отвечать	1<br>2<br>3<br>4<br>5<br>6<br>7<br>88	C5

17	Каково Ваше семейное положение?	Холост/не замужем 1<br>Женат/ замужем 2<br>Женат/ замужем, но живем раздельно 3<br>Разведен/а 4<br>Вдовец/вдова 5<br>В гражданском браке 6	C7
18	Что из перечисленного более всего соответствует Вашему <u>основному</u> роду занятий за последние 12 месяцев?<br><br>(ИСПОЛЬЗУЙТЕ КАРТОЧКУ ОТВЕТОВ)	Государственный служащий 1<br>Работник частного сектора 2<br>Предприниматель 3<br>Неоплачиваемый труд 4<br>Учащийся 5<br>Домохозяйка 6<br>Пенсионер 7<br>Безработный (способный работать) 8<br>Безработный (неспособный работать) 9<br>Отказывается отвечать 88	C8
19	Сколько человек, <u>включая Вас</u>, проживают вместе с Вами?<br>В том числе старше 18 лет?	Количество человек └─┴─┘<br>Количество человек старше 18 лет └─┴─┘	C9

**РАСШИРЕННЫЙ МОДУЛЬ: Демографическая информация,** продолжение			
	**Вопросы**	**Ответы**	**Код**
20	Каков средний уровень доходов Вашей семьи за последний год в тенге?<br><br>*(УКАЖИТЕ ТОЛЬКО ОДИН ВАРИАНТ, НЕ ВСЕ 3)*	В неделю └─┴─┴─┴─┴─┴─┴─┘ Перейдите к *T1*	C10a
		ИЛИ в месяц └─┴─┴─┴─┴─┴─┴─┘ Перейдите к *T1*	C10b
		ИЛИ в год └─┴─┴─┴─┴─┴─┴─┘ Перейдите к *T1*	C10c
		Отказывается отвечать 8	C10d
21	Если Вы не знаете точную сумму, можете ли Вы приблизительно оценить уровень месячного дохода на одного члена семьи, если я предложу Вам некоторые возможные варианты?	≤ 25 000 тенге 1<br>Больше, чем 25 000, ≤ 2<br>Больше, чем 35 000, ≤ 3<br>Больше, чем 50 000, ≤ 4<br>Больше, чем 70 000 5<br>Не знаю 7<br>Отказывается 8	C11

Этап 1 Показатели поведения

**ОСНОВНОЙ МОДУЛЬ: Употребление табака**			
Теперь я задам Вам несколько вопросов в отношении форм поведения, влияющих на состояние здоровья, таких как курение, употребление алкоголя, потребление фруктов и овощей и физическая активность.			
	**Вопросы**	**Ответ**	**Код**
22	Употребляете ли Вы в настоящее время какие-либо табачные изделия, например, сигареты, сигары или курите трубку?	Да 1<br>Нет *2*<br>*Если нет, перейдите к T6*	T1
23	Употребляете ли Вы табачные изделия ежедневно?	Да 1<br>Нет 2<br>*Если нет, перейдите к T6*	T2
24	В каком возрасте Вы начали курить ежедневно?	Возраст (годы) └─┴─┴─┘<br>Не помню 777 *Если известно, перейдите к T5a*	T3
25	Помните ли Вы, как давно это было?<br><br>*(УКАЖИТЕ ТОЛЬКО ОДИН ВАРИАНТ, НЕ ВСЕ 3)*<br><br>Затруднен 777	Сколько лет назад └─┴─┴─┘ *Если известно, перейдите к T5a*	T4a
		ИЛИ месяцев └─┴─┴─┘ *Если известно, перейдите к T5a*	T4b
		ИЛИ недель	T4c

26	Какое количество следующих табачных изделий, в среднем, Вы употребляете ежедневно?<br><br>*(УКАЖИТЕ КОЛ-ВО ПО КАЖДОМУ ВИДУ ИЗДЕЛИЙ) (ИСПОЛЬЗУЙТЕ КАРТОЧКУ ОТВЕТОВ)*<br><br>Не помню 777	Сигареты/папиросы фабричного производства	└─┴─┴─┘	T5a
		Сигареты папиросы, скрученные вручную	└─┴─┴─┘	T5b
		Трубки, набитые табаком	└─┴─┴─┘	T5c
		Различные типы сигар	└─┴─┴─┘	T5d
		Другое	└─┴─┴─┘<br>*Если другое, перейдите к T5 other, иначе - к T9*	T5e
		Другое (укажите):	*Перейдите к T9*	T5 other

РАСШИРЕННЫЙ МОДУЛЬ: Употребление табака, продолжение

Вопросы		Ответ		Код
27	Курили ли Вы <u>ежедневно когда-либо</u> ранее?	Да	1	T6
		Нет	2 *Если нет, перейдите к T9*	
28	В каком возрасте Вы <u>прекратили</u> курить <u>ежедневно?</u>	Возраст (годы)	└─┴─┴─┘ *Если известно, перейдите к T9*	T7
		Не помню 777		
29	<u>Как давно</u> Вы прекратили курить ежедневно?<br><br>*(УКАЖИТЕ ТОЛЬКО ОДИН ВАРИАНТ, НЕ ВСЕ 3)*<br><br>Не помню 777	лет назад	└─┴─┴─┘ *Если известно, перейдите к*	T8a
		ИЛИ месяцев назад	└─┴─┘ *Если известно, перейдите к T9*	T8b
		ИЛИ недель назад	└─┴─┘	T8c
30	Употребляете ли <u>Вы в настоящее время</u> какие-либо <u>другие табачные</u> [illegible]	Да	1	T9
		Нет	2 *Если нет,*	

ОСНОВНОЙ МОДУЛЬ: Употребление алкоголя

Следующие вопросы касаются употребления алкоголя.

Вопросы		Ответ		Код
31	Употребляли ли Вы когда-либо алкоголь (например, пиво, вино, водка и другие крепкие напитки)	Да	1	A1
		Нет	2 *Если нет, перейдите к D1*	
32	Употребляли ли Вы алкоголь (например, пиво, вино, крепкие напитки) в течение <u>последних 12 месяцев?</u>	Да	1 *Если да, перейдите к A2*	A1б
		Нет	2	

33	Прекращали ли Вы употребление алкоголя по причине его негативного влияния на Ваше здоровье или по совету врача или другого медицинского работника?	Да<br>Нет	1 *перейдите к D1*<br>2 *перейдите к D1*	A1в
34	За последние 12 месяцев, как часто Вы выпивали хотя бы одну стандартную дозу алкоголя?<br>*(ПРОЧИТАЙТЕ ОТВЕТЫ ИСПОЛЬЗУЙТЕ КАРТОЧКУ ОТВЕТОВ)*	Ежедневно<br>5-6 дней в неделю<br>1-4 дня в неделю<br>1-3 дня в месяц<br>Менее одного раза в месяц	1<br>2<br>3<br>4<br>5	A2
35	Употребляли ли Вы алкоголь за последние 30 дней?	Да<br>Нет	1<br>2 *Если нет, перейдите к D1*	A3
36	За последние 30 дней, сколько раз Вы выпивали хотя бы по 1 стандартной дозе алкоголя?	Количество<br>Не знаю 77	└─┴─┘	A4
37	За последние 30 дней, когда Вы выпивали, сколько стандартных доз любого алкогольного напитка в среднем (бокалов/стаканов/рюмок) Вы выпивали в каждом случае?	Количество<br>Не знаю 77	└─┴─┘	A5
38	Какое наибольшее количество стандартных доз алкоголя Вы принимали за один случай в течение последних 30 дней, учитывая все виды алкогольных напитков вместе?	Наибольшее количество<br>Не знаю 77	└─┴─┘	A6
39	За последние 30 дней сколько раз Вы выпивали 6 и более стандартных доз за 1 случай?	Сколько раз<br>Не знаю 77	└─┴─┘	A7
40	За последние 30 дней, когда Вы выпивали, сколько раз это сопровождалось приемом пищи?	Обычно с приемом пищи<br>Иногда с приемом пищи<br>Редко с приемом пищи<br>Никогда	1<br>2<br>3<br>4	A8
41	Сколько стандартных доз любого алкогольного напитка Вы употребляли в течение каждого дня за последние 7 дней?	Понедельник<br>Вторник<br>Среда<br>Четверг	└─┴─┘<br>└─┴─┘<br>└─┴─┘<br>└─┴─┘	A9

		Пятница └─┴─┘	
		Суббота └─┴─┘	
	(ИСПОЛЬЗУЙТЕ КАРТОЧКИ С ОТВЕТАМИ)	Воскресенье └─┴─┘	

**ОСНОВНОЙ МОДУЛЬ: Рацион питания**			
Следующие вопросы касаются потребления фруктов и овощей в обычном рационе питания. Некоторые примеры местных фруктов и овощей изображены на карте питания. Размер порции показан на картинке. Представьте данные о питании в течение обычной недели за последний год.			
**Вопросы**		**Ответ**	**Код**
42	Сколько дней в типичную неделю Вы обычно потребляете фрукты?	Количество дней └─┴─┘ *Если 0 дней, перейдите к D3*<br>Не знаю 77	D1
43	Сколько порций фруктов Вы потребляете за один из таких дней?	Количество порций └─┴─┘<br>Не знаю 77	D2
44	Сколько дней в типичную неделю Вы обычно потребляете овощи?	Количество дней └─┴─┘ *Если 0 дней, перейдите к D5*<br>Не знаю 77	D3
45	Сколько порций овощей Вы потребляете за один из таких дней?	Количество порций └─┴─┘<br>Не знаю 77	D4
**Пищевая соль**			
В этом разделе мы хотели бы узнать о количестве соли в вашем рационе. Пищевая соль включает в себя обычную поваренную соль, неочищенную соль и йодированную соль, соленые бульонные кубики и порошки, а также соленые соусы, такие как соевый или рыбный соусы (см. карточки). Следующие вопросы посвящены добавлению соли в пищу непосредственно перед употреблением, тому, как Вы готовите дома, об употреблении обработанных продуктов с высоким содержанием соли, например (впишите примеры продуктов, специфичных для Вашей страны), а так же о том, как вы контролируете потребление соли.<br>Пожалуйста, ответьте на следующие вопросы, даже если Вы считаете, что Ваш рацион содержит мало соли.			
46	Как часто Вы добавляете соль или соленые соусы в пищу перед ее употреблением или непосредственно во время еды?<br>(Выберите только один ответ)	Всегда 1<br>Часто 2<br>Иногда 3<br>Редко 4<br>Никогда 5<br>Не знаю 77	D5
47	Как часто соль, соленые специи или соленые соусы добавляются во время приготовления пищи у Вас в быту?	Всегда 1<br>Часто 2<br>Иногда 3<br>Редко 4<br>Никогда 5<br>Не знаю 77	D6

48	Как часто Вы употребляете обработанные продукты с высоким содержанием соли?<br>*Под обработанными продуктами с высоким содержанием соли я имею в виду продукты, которые изменены по сравнению с их естественным состоянием, например, мясные и рыбные копчености, колбаса, сало, соленья, консервы, соленая сюзьма, соленый курут, соленые чипсы и орехи.*<br>(Используйте карточки с ответами)	Всегда 1<br>Часто 2<br>Иногда 3<br>Редко 4<br>Никогда 5<br>Не знаю 77	D7
49	Как Вы думаете, какое количество соли или соленых соусов Вы употребляете?	Слишком<br>много 1<br>Много 2<br>Среднее 3<br>количество 4<br>Мало 5<br>Слишком мало 77<br>Не знаю	D8

ОСНОВНОЙ МОДУЛЬ: Физическая активность

Теперь я намерен спросить, сколько времени в неделю Вы обычно уделяете различным видам физической активности. Просьба ответить на эти вопросы, даже если не считаете себя физически активным человеком. Ответ должен включать различные виды деятельности - работу, уход за домом и садом, передвижение с одного места на другое (связанное с использованием транспорта), а также активный досуг (в свободное от работы время или на отдыхе) или занятия спортом. Эту вступительную часть **не следует** пропускать.

Активность на работе

	**Вопросы**	**Ответ**	**Код**
50	Требует ли Ваша работа высокоинтенсивной деятельности, при которой значительно учащается дыхание или пульс [*например, поднятие тяжестей, земляные или строительные работы*] непрерывно в течение, по меньшей мере, 10 минут?<br>*(ИСПОЛЬЗУЙТЕ КАРТОЧКУ ОТВЕТОВ)*	Да 1<br>Нет 2<br>*Если нет, перейдите к P 4*	P1
51	Сколько дней в неделю Вы обычно занимаетесь высокоинтенсивной деятельностью на работе?	Количество дней └─┘	P2
52	Сколько часов в день Вы обычно занимаетесь высокоинтенсивной деятельностью на работе?	Часы : минуты └─┴─┘ : └─┴─┘<br>часов минут	P3 (a-b)
53	Требует ли Ваша работа среднеинтенсивной деятельности, при	Да 1	P4

	которой немного учащается дыхание или пульс *[например, быстрая ходьба или поднятие небольших тяжестей*] непрерывно в течение, по меньшей мере, 10 минут?	Нет 2<br>*Если нет, перейдите к Р 7*	
54	Сколько дней в неделю Вы обычно занимаетесь среднеинтенсивной деятельностью на работе?	Количество дней └─┘	P5
55	Сколько часов в день Вы обычно занимаетесь среднеинтенсивной деятельностью на работе?	Часы : минуты └─┴─┘ : └─┴─┘<br>часов минут	P6 (a-b)
**Передвижение**			
Следующие вопросы исключают физическую нагрузку на работе, у которой уже говорилось. Теперь хотелось бы узнать о том, как Вы обычно передвигаетесь, например по пути на работу, в магазины, на рынок, в мечеть [если необходимо, приведите другие примеры]			
**Вопросы**		**Ответ**	**Код**
56	Используете ли Вы ходьбу пешком или езду на велосипеде непрерывно в течение не менее 10 минут в качестве средства передвижения?	Да 1<br>Нет 2 *Если нет, перейдите к Р 10*	P7
57	Сколько дней в неделю Вы обычно ходите пешком или ездите на велосипеде непрерывно в течение не менее 10 минут?	Количество дней └─┘	P8
58	Сколько часов в день Вы обычно уделяете ходьбе пешком или езде на велосипеде в день?	Часы : минуты └─┴─┘ : └─┴─┘<br>часов минут	P9 (a-b)
**Активный досуг**			
Следующие вопросы исключают деятельность, связанную с работой и передвижением, о которой уже говорилось. Теперь мне хотелось бы узнать о занятиях спортом, физическими упражнениями и активным досугом [добавьте необходимое].			
**Вопросы**		**Ответ**	**Код**
59	Носят ли Ваши занятия спортом, физическими упражнениями или активным досугом высокоинтенсивный характер, при котором значительно учащается дыхание или пульс *[например, бег или футбол*] непрерывно в течение, по меньшей мере, 10 минут? *(ИСПОЛЬЗУЙТЕ КАРТОЧКУ ОТВЕТОВ)*	Да 1<br>Нет 2<br>*Если нет, перейдите к Р 13*	P10
60	Сколько дней в неделю Вы обычно занимаетесь спортом, физическими упражнениями или активным досугом высокоинтенсивного характера?	Количество дней └─┘	P11
61	Сколько часов в день Вы обычно занимаетесь спортом, физическими упражнениями или активным досугом высокоинтенсивного характера?	Часы : минуты └─┴─┘ : └─┴─┘<br>часов минут	P12 (a-b)

62	Носят ли Ваши занятия спортом, физическими упражнениями или активным досугом среднеинтенсивный характер, при котором незначительно учащается дыхание или пульс *[например, быстрая ходьба, езда на велосипеде, плавание, волейбол]* непрерывно в течение, по меньшей мере, 10 минут?	Да 1<br>Нет 2<br>*Если нет, перейдите к H1*	P13
63	Сколько дней в неделю Вы обычно занимаетесь спортом, физическими упражнениями или активным досугом среднеинтенсивного характера?	Количество дней └─┘	P14
64	Сколько часов в день Вы обычно занимаетесь спортом, физическими упражнениями или активным досугом <u>среднеинтенсивного</u> характера?	Часы : минуты └─┴─┘ : └─┴─┘<br>часов минут	P15 (a-b)

**РАСШИРЕННЫЙ МОДУЛЬ: История повышенного артериального давления**			
**Вопросы**		**Ответы**	
65	Измерял ли Вам артериальное давление врач или другой медицинский работник когда-нибудь?	Да 1<br>Нет 2<br>Если нет, перейдите к H6	H1
66	Говорил ли Вам когда-либо врач или другой медицинский работник, что у Вас повышенное артериальное давление или гипертония?	Да 1<br>Нет 2<br>Если нет, перейдите к H6	H2a
67	Говорил ли Вам врач или другой медицинский работник в течение последних 12 месяцев, что у Вас повышенное артериальное давление или гипертония?	Да 1<br>Нет 2	H2b
68	В течение последних 2 недель принимали ли Вы какие-либо лекарства от повышенного артериального давления, выписанные врачом или другим медицинским сотрудником?	Да 1<br>Нет 2	H2c
69	Обращались ли Вы когда либо к народным целителям по поводу повышенного артериального давления или гипертонии?	Да 1<br>Нет 2	H2d
70	Принимаете ли Вы в настоящее время какие-либо лекарственные травы или народные средства для снижения артериального давления?	Да 1<br>Нет 2	H2e

71	Если у Вас было повышенное давление, то какими были максимальные показатели?	Систолическое (мм рт.ст.) └─┴─┴─┘<br>Диастолическое (мм рт.ст.) └─┴─┴─┘	H2f
72	Получаете ли Вы в настоящее время какой-либо из перечисленных ниже рекомендаций по поводу высокого артериального давления по назначению врача или другого медицинского работника?		
	Рекомендации по ограничению употребления соли в еде	Да 1<br>Нет 2	H3a
	Рекомендации или лечебные процедуры для снижения веса	Да 1<br>Нет 2	H3b
	Рекомендации или лечебные процедуры для отказа от курения	Да 1<br>Нет 2	H3c
	Рекомендации начать заниматься физическими упражнениями или увеличить физические нагрузки	Да 1<br>Нет 2	H3d
73	Состоите ли вы на диспансерном учете по поводу высокого артериального давления	Да 1<br>Нет 2	H3e
74	Принимаете ли Вы в настоящее время какие-либо лекарства (медицинские препараты) от повышенного давления и если да, то когда Вы это делаете?	Вообще не принимаю 1<br>Принимаю ежедневно вне зависимости от давления 2<br>Принимаю только при повышенном кровяном давлении 3<br>Принимаю только при кризах 4	H4

**ОСНОВНОЙ МОДУЛЬ: История Диабета**			
**Вопросы**		**Ответы**	**Код**
75	Измерялся ли когда-либо уровень сахара в Вашей крови медицинским работником?	Да 1<br>Нет 2 Если нет, перейдите к L1	H6
76	Говорил ли Вам когда-либо медицинский работник, что у Вас повышен уровень сахара в крови, либо что Вы страдаете диабетом?	Да 1<br>Нет 2 Если нет, перейдите к L1	H7a
77	Говорил ли Вам врач или другой медицинский работник в течение последних 12 месяцев, что у Вас повышен уровень сахара в крови, либо что Вы страдаете диабетом?	Да 1<br>Нет 2	H7b
78	В течение последних 2 недель принимали ли Вы какие-либо лекарства (медицинские препараты) от диабета, выписанные врачом или другим медицинским сотрудником?	Да 1<br>Нет 2	H8
79	Принимаете ли Вы в настоящее	Да 1	H9

	время инсулин для лечения диабета, назначенный врачом или другим медицинским работником?	Нет 2	
80	Получаете ли Вы в настоящее время какой-либо из перечисленных рекомендаций по поводу диабета по назначению врача или другого медицинского работника?		H10
	Специально назначенная диета	Да 1<br>Нет 2	
	Рекомендации или лечебные процедуры для снижения веса	Да 1<br>Нет 2	
	Рекомендации или лечебные процедуры для отказа от курения	Да 1<br>Нет 2	
	Рекомендации начать заниматься физическими упражнениями или увеличить нагрузки	Да 1<br>Нет 2	
81	Состоите ли вы на диспансерном учете по поводу сахарного диабета?	Да 1<br>Нет 2	H11
82	Есть ли у Вас осложнения сахарного диабета?	Да 1<br>Нет 2 Перейдите к H14	H12
83	Если Да, то какие осложнения?	диабетическая ретинопатия 1<br>диабетическая нефропатия 2<br>полинейропатия 3<br>периферическая ангиопатия 4<br>диабетическая стопа (язвы, деформации стоп) 5	H13
84	Бывают ли у Вас гипогликемические состояния?	Да 1<br>Нет 2 Перейдите к H16	H14
85	Если Да, то как часто?	Каждую неделю 1<br>2-3 раза в месяц 2<br>Несколько раз в год 3	H15
86	Обращались ли Вы за последние 12 месяцев к народным целителям по поводу диабета?	Да 1<br>Нет 2	H16
87	Принимаете ли Вы в настоящее время какие-либо лекарственные травы или народные средства для лечения диабета?	Да 1<br>Нет 2	H17

**РАСШИРЕННЫЙ МОДУЛЬ: История повышенного общего холестерина**		
**Вопросы**	**Ответы**	**Код**

88	Измерял ли Вам врач или другой медицинский работник когда-либо уровень холестерина (уровень жиров в крови)?	да 1<br>нет 2 Перейдите к L7	L1
89	Говорил ли Вам когда-либо врач или другой<br>медицинский работник, что у Вас повышенный уровень холестерина?	да 1<br>нет 2 Перейдите к L7	L2
90	За последние 12 месяцев говорил ли Вам врач или другой медицинский работник, что у Вас повышенный уровень холестерина?	да 1<br>нет 2	L3
91	В течение последних 2 недель принимали ли Вы препараты (лекарства) от повышенного холестерина, выписанные врачом или другим медицинским работником?	да 1<br>нет 2	L4
92	Обращались ли Вы когда-либо к народным целителям по поводу повышенного холестерина?	да 1<br>нет 2	L5
93	Принимаете ли Вы в настоящее время какие-либо<br>лекарственные травы или народные средства для<br>лечения повышенного холестерина?	да 1<br>нет 2	L6

**РАСШИРЕННЫЙ МОДУЛЬ: Наследственность по заболеваниям**			
**Вопросы**		**Ответы**	**Код**
94	Есть ли у вас родственники первой степени родства (родители, родные братья и сестры)		L7
	Болевшие гипертонией (повышенным кровяным давлением)?	да 1<br>нет 2	
	Перенесшие инфаркт миокарда или инсульт в возрасте моложе 55 лет для мужчин и 65 лет для женщин?	да 1<br>нет 2	
	Болевшие сахарным диабетом?	да 1<br>нет 2	
	Имевшие злокачественные опухоли органов пищеварения (желудка, кишечника, печени, поджелудочной железы)?<br>Если да, то уточните локализацию.	да 1<br>нет 2<br>____________________<br>__	

	Болевшие вирусным гепатитом, циррозом печени, язвенной болезнью, язвенным колитом, болезнью Крона?<br>Если да, то уточните какими заболеваниями болели?	да 1<br>нет 2<br>______________________	
	Болевшие болезнью обмена веществ (болезнь Вильсона – Коновалова, гемохроматоза, гемосидероз)?<br>Если да, то уточните какими заболеваниями болели?	да 1<br>нет 2<br>______________________	
	Болевшие бронхиальной астмой или аллергией любого типа?	да 1<br>нет 2	
	Имеющие заболевания почек?	да 1<br>нет 2	
	Болевшие анемией?	да 1<br>нет 2	
	Болевшие хроническим бронхитом?	да 1<br>нет 2	

**РАСШИРЕННЫЙ МОДУЛЬ: История сердечно-сосудистых заболеваний**			
**Вопросы**		**Ответы**	**Код**
95	Говорил ли Вам **когда-либо врач** или другой медицинский работник, что у Вас есть какие-либо сердечно-сосудистые заболевания?	Да 1<br>Нет 2 перейдите к S8	S1
96	Какие сердечно-сосудистые заболевания сердца были когда-либо у Вас диагностированы?		S2
	Ишемическая болезнь сердца	да 1<br>нет 2	
	Стенокардия	да 1<br>нет 2	
	Острое нарушение мозгового кровообращения (инсульт)	да 1<br>нет 2	
	Инфаркт миокарда	да 1<br>нет 2	
	Хроническая сердечная недостаточность	да 1<br>нет 2	
	Мерцательная аритмия (фибрилляция предсердий)	да 1<br>нет 2	
	Трепетание предсердий	да 1<br>нет 2	
97	Состоите ли Вы на диспансерном учете по поводу какого-либо сердечно-сосудистого заболевания	да 1<br>нет 2 Перейдите к S5	S3

98	Укажите, по поводу какого сердечно-сосудистого заболевания Вы состоите на диспансерном учете	____________________ ____	S4
99	Госпитализировали ли Вас по поводу заболевания сердца в течение последних 12 месяцев?	да 1<br>нет 2 Перейдите к S7	S5
100	Если да, то с каким диагнозом Вас госпитализировали?	____________________ ____	S6
101	Проводились ли Вам когда-либо следующие диагностические и операционные процедуры?		S7
	Нагрузочные электро-кардиографические (ЭКГ) тесты?	да 1<br>нет 2	
	Коронароангиография	да 1<br>нет 2	
	Стентирование	да 1<br>нет 2	
	Аортокоронарное шунтирование	да 1<br>нет 2	

**РАСШИРЕННЫЙ МОДУЛЬ: Симптомы сердечно-сосудистых заболеваний**			
**Вопросы**		**Ответы**	**Код**
102	Беспокоят ли Вас хотя бы время от времени следующие из перечисленного?		S8
	Перебои в работе сердца	Да 1<br>Нет 2	
	Приступы учащенного сердцебиения	Да 1<br>Нет 2	
	Приступы головокружения, внезапной слабости, внезапной потери сознания	Да 1<br>Нет 2	
	Ощущения остановки сердца	Да 1<br>Нет 2	
103	Бывает ли у Вас усталость, одышка, или учащенное сердцебиение?	При физической нагрузке средней интенсивности 1<br>При малейшей физической нагрузке 2<br>3<br>Возникает даже в состоянии покоя 4<br>Не бывает одышки	S9
104	Бывают ли у Вас жгучие, давящие боли, дискомфорт за грудиной, в левой руке, в левой половине груди?	Да 1<br>Нет 2 Перейти к S14	S10
105	Если возникают боли, то в каких ситуациях чаще всего?	В покое 1<br>При эмоциональной нагрузке 2<br>При физической нагрузке (подъем в гору, по лестнице, быстрая ходьба) 3	S11
106	Отметьте продолжительность боли по времени	5 – 10 минут 1<br>Более 10 минут 2	S12

107	Что вы делаете, чтобы снять боль?	Принимаю нитроглицерин 1<br>Прекращаю нагрузку (останавливаюсь) 2<br>Продолжаю идти, не снижая темпа 3	S13

РАСШИРЕННЫЙ МОДУЛЬ: Вирусные гепатиты

	Вопросы	Ответы	Код
108	Обследовались ли Вы когда-либо на вирусные гепатиты В, С, D? Если, то каким был результат?		S14

	Обследовались		Результат		
	Да	Нет	Положительный	Отрицательный	Не знаю
Вирусный гепатит В	1	2	1	2	3
Вирусный гепатит С	1	2	1	2	3
Вирусный гепатит D	1	2	1	2	3

	Вопросы	Ответы	Код
109	Был ли у Вас в жизни эпизод острого гепатита (болезнь Боткина)?	Да 1<br>Нет 2	S15
110	Болеете ли Вы гемофилией или другими онкогематологическими заболеваниями?	Да 1<br>Нет 2	S16
111	Находитесь ли Вы на гемодиализе?	Да 1<br>Нет 2	S17
112	Переливали ли Вам кровь?	Да 1<br>Нет 2	S18
113	Употребляли ли Вы наркотики путем инъекций?	Да 1<br>Нет 2	S19
114	Болеет ли кто-либо в Вашей семье вирусным гепатитом?	Да 1<br>Нет 2	S20
115	Проводились ли Вам оперативные вмешательства (операции)?	Да 1<br>Нет 2	S21
116	Делали ли Вам татуировки и/или пирсинг?	Да 1<br>Нет 2	S22
117	Вакцинировались ли Вы от гепатита В?	Да 1<br>Нет 2	S23

РАСШИРЕННЫЙ МОДУЛЬ: Болезни органов дыхания

	Вопросы	Ответы	Код
118	Говорил ли Вам когда-либо медицинский работник, что у Вас один из перечисленных диагнозов?	Бронхиальная астма 1<br>Хронический бронхит 2<br>Хронический ринит (поллиноз, риносинусит, полипоз носа) 3	S24

		Хроническая обструктивная болезнь легких 4<br>Ничего из вышеперечисленного 5	
119	Беспокоит ли Вас кашель с мокротой вне простудных заболеваний?	Да 1<br>Нет 2	S25
120	Беспокоит ли Вас кашель с мокротой по утрам?	Да 1<br>Нет 2	S26
121	Провоцирует ли плохая погода у Вас кашель?	Да 1<br>Нет 2	S27
122	Как часто у Вас возникает одышка?	Никогда 1<br>Иногда 2<br>Часто 3	S28
123	Бывала ли у Вас когда-либо аллергия?	Да 1<br>Нет 2	S29
124	Переносили ли Вы операции на носовой полости?	Да 1<br>Нет 2	S30
125	Отмечали ли Вы приступы нехватки воздуха (или удушья или сильного кашля)?	Да 1<br>Нет 2 перейти к S34	S31
126	Страдаете ли Вы от ночных приступов нехватки воздуха (или удушья, или сильного кашля)?	Да 1<br>Нет 2 перейти к S34	S32
127	Если да, то сколько эпизодов в неделю у Вас бывает?	└─┴─┴─┘	S33

**РАСШИРЕННЫЙ МОДУЛЬ: Ревматология**			
**Вопросы**		**Ответы**	**Код**
128	Болят ли у Вас суставы?	Да 1<br>Нет 2 Перейдите к S37	S34
129	Какие суставы у вас болят?<br><br>*<u>Крупные:</u> коленные, тазобедренные, лучезапястные, голеностопные*<br>*<u>Мелкие:</u> пястно-фаланговые, межфаланговые, кистей, плюснефаланговые стоп*	Крупные 1<br>Мелкие 2	S35
130	В какое время суток, в основном болят суставы?	Утром 1<br>Вечером 2	S36
131	Припухают ли у Вас суставы?	Да 1<br>Нет 2 Перейдите к S40	S37

132	Если да, то какие суставы припухают?<br><br>*<u>Крупные:</u> коленные, тазобедренные, лучезапястные, голеностопные*<br><br>*<u>Мелкие:</u> пястно-фаланговые, межфаланговые кистей, плюснефаланговые стоп*	Крупные<br>Мелкие	1<br>2	S38
133	Как часто припухают суставы?	Время от времени<br>Постоянно	1<br>2	S39
134	Ощущаете ли Вы утреннюю скованность в суставах?	Да<br>Нет	1<br>2 Перейдите к S43	S40
135	Если да, то в каких суставах?	Крупные<br>Мелкие	1<br>2	S41
136	Если да, то какая продолжительность скованности?	менее 30 минут<br>более 30 минут	1<br>2	S42
137	Появляются ли у Вас боли при движении, ходьбе, поднятии на лестницу, или спуске с лестницы?	Да<br>Нет	1<br>2	S43
138	Появляется ли у Вас хруст при движении суставов?	Да<br>Нет	1<br>2	S44
139	Появляются ли у Вас «заклинивания в суставах», затруднения, тугоподвижность в суставах при движении?	Да<br>Нет	1<br>2	S45
140	Обращались ли Вы к врачам по поводу болей или припухлости в суставах?	Да<br>Нет	1<br>2	S46
141	Состоите ли Вы на учете у ревматолога или терапевта по поводу заболеваний суставов: остеоартроза или ревматоидного артрита?	Да<br>Нет	1<br>2	S47
142	Появляется ли боль при сжатии кисти?	Да<br>Нет	1<br>2	S48

**РАСШИРЕННЫЙ МОДУЛЬ: Анемия**				
**Вопросы**		**Ответы**		**Код**
143	Говорил ли Вам когда-либо медицинский работник, что у Вас анемия?	Да<br>Нет	1<br>2 Перейдите к S51	S49
144	Получали Вы лечение по поводу анемии?	Да<br>Нет	1<br>2	S50
145	Бывают ли у Вас частые носовые кровотечения (1-2 раза в неделю)	Да<br>Нет	1<br>2	S51
146	Для женщин фертильного возраста	Да	1	S52

	(18-49 лет): Наблюдаются ли у вас обильные и длительные месячные?	Нет 2	
147	Отмечали ли Вы когда-либо у себя черный цвет стула?	Да 1<br>Нет 2	S53
148	Принимали ли Вы когда-нибудь препараты железа?	Да 1<br>Нет 2	S54

**РАСШИРЕННЫЙ МОДУЛЬ: Хронические болезни почек**			
**Вопросы**		**Ответы**	**Код**
149	Говорил ли Вам **когда-либо** врач или другой медицинский работник, что у Вас заболевание почек?	Да 1<br>Нет 2 перейти к S58	S55
150	Говорил ли Вам **когда-либо** врач или другой медицинский работник, что у Вас один из перечисленных диагнозов?	Пиелонефрит 1<br>Гломерулонефрит 2<br>Мочекаменная болезнь 3<br>Хроническая почечная недостаточность 4	S56
151	Состоите ли Вы на диспансерном учете по поводу заболеваний почек?	Да 1<br>Нет 2	S57
152	Беспокоят ли Вас хотя бы время от времени следующие из перечисленного :	Отеки 1<br>Головная боль 2<br>Боли в спине 3<br>Слабость 4<br>Жажда 5<br>Полиурия 6<br>Красная моча 7<br>Мало мочи 8<br>Болезненное мочеиспускание 9	S58

**РАСШИРЕННЫЙ МОДУЛЬ: Медикаменты**			
**Вопросы**		**Ответы**	**Код**
153	Принимали ли Вы в течение последних 30 дней какие-либо лекарственные препараты/медикаменты?	да 1<br>нет 2 Перейдите к S62	S59
154	Если Да, укажите, пожалуйста, какие препараты из этого списка Вы принимали:	Аспирин или др. Антиагреганты 1<br>Антикоагулянты 2<br>Варфарин 2.1<br>Новые оральные антикоагулянты 2.2<br>Бета-блокаторы Ингибиторы АПФ 3<br>Блокаторы рецепторов АТ-II 4	S60

		Блокаторы кальциевых каналов 5<br>Диуретики 6<br>Другие анти-гипертензивные препараты 7<br>Антиаритмические 8<br>Статины 9<br>Инсулин 10<br>Пероральные сахароснижающие препараты 11<br>Ингаляторы 12	
155	За последний месяц, пропускали ли Вы прием лекарств или изменяли ли дозу назначенных врачом препаратов самостоятельно?	Да 1<br>Нет 2	S61
156	Если вы принимаете варфарин, определяете ли Вы МНО?	Да 1<br>Нет 2	S62
157	Если вы принимаете варфарин, то какой уровень МНО Вы поддерживаете?	Менее 2 1<br>2-3 2<br>Более 3 3<br>Не знаю 4	S63

**РАСШИРЕННЫЙ МОДУЛЬ: Советы по здоровому образу жизни**			
В течение последних трех лет, рекомендовал ли врач или другой медицинский работник Вам делать что-либо из нижеперечисленного?			
**Вопросы**		**Ответы**	**Код**
158	Прекратить употребление табачной продукции или вовсе не начинать курить	Да 1<br>Нет 2	S62
	Уменьшить употребление соли в еде	Да 1<br>Нет 2	
	Ежедневно употреблять минимум пять порций фруктов и/или овощей	Да 1<br>Нет 2	
	Уменьшить употребление жиров в еде	Да 1<br>Нет 2	
	Начать заниматься спортом или увеличить количество физической активности	Да 1<br>Нет 2	
	Поддерживать нормальный (здоровый) вес тела или похудеть	Да 1<br>Нет 2	

Этап 2 Данные физического осмотра

ОСНОВНОЙ МОДУЛЬ: Артериальное давление			
1	Идентификатор лица, измеряющего артериальное давление	└─┴─┴─┘	M1
2	Идентификатор инструмента для измерения артериального давления	└─┴─┘	M2
3	Размер манжетки тонометра	Маленький 1<br>Средний 2<br>Большой 3	M3
4	Показание 1	Систолическое (мм рт.ст.) └─┴─┴─┘	M4
		Диастолическое (мм рт.ст.) └─┴─┴─┘	M5
5	Показание 2	Систолическое (└─┴─┴─┘	M6
		Диастолическое (мм рт.ст.) └─┴─┴─┘	M7
6	Показание 3	Систолическое (мм рт.ст.) └─┴─┴─┘	M8
		Диастолическое (мм рт.ст.) └─┴─┴─┘	M9

ОСНОВНОЙ МОДУЛЬ: Рост и вес

Вопросы		Ответ	Код
7	(Для женщин) Вы беременны?	Да 1<br>Нет 2	M10
8	Идентификатор лица, измеряющего рост и вес	└─┴─┴─┘	M11
9	Идентификатор устройства для измерения роста и веса	Рост └─┴─┘	M12a
		Вес └─┴─┘	M12b
10	Рост	сантиметры (см) └─┴─┴─┘.└─┘	M13
11	Вес<br>*Если слишком большой вес, код 666.6*	килограммы (кг) └─┴─┴─┘.└─┘	M14

ОСНОВНОЙ МОДУЛЬ: Талия

12	Идентификатор устройства для измерения талии	└─┴─┘	M15
13	Окружность талии	сантиметры (см) └─┴─┴─┘.└─┘	M16

РАСШИРЕННЫЙ МОДУЛЬ: Пикфлоуметрия и спирометрия

Вопросы	Ответ	Код

14. Значение пикфлоуметрии (для лиц 40 лет и старше)		M17
15. Значение спирометрии		M18

Этап 3 Биохимические показатели

ОСНОВНОЙ МОДУЛЬ: Уровень глюкозы в крови			
**Вопросы**		**Ответ**	**Код**
16	Принимали ли Вы за последние 12 часов какую-нибудь пищу или пили ли что-нибудь, кроме воды?	Да 1<br>Нет 2	B1
17	Идентификатор лаборанта	└─┴─┴─┘	B2
18	В какое время дня был взят анализ (по 24-часовой шкале)	Часы : минуты └─┴─┘ : └─┴─┘<br>часов минут	B3
19	Уровень глюкозы в крови натощак	ммоль/л └─┴─┘ . └─┴─┘	B4
20	Принимали ли Вы <u>сегодня и</u>нсулин или какие-либо лекарственные средства, назначенные врачем или другим медицинским работником для лечения повышенного уровня сахара в крови?	Да 1<br>Нет 2	B5

ОСНОВНОЙ МОДУЛЬ: Липиды крови			
21	Общий холестерин	ммоль/л └─┴─┘ . └─┴─┘	B6
22	Принимали ли Вы лекарства (медицинские препараты) для снижения уровня холестерола в крови по назначению врача или другого медицинского работников за последние две недели?	Да 1<br>Нет 2	B7

ОСНОВНОЙ МОДУЛЬ: Натрий и креатинин в моче			
23	Соблюдали ли Вы пост перед сдачей мочи?	Да 1<br>Нет 2	B8
24	Идентификатор лаборанта	└─┴─┘ .	B9
25	Время сутки выборки пробы мочи (по 24-часовой шкале)	└─┴─┘ : └─┴─┘<br>часов минут	B10
26	Натрий в моче	└┴┴┘ └┘ Ммоль/л	B11
27	Креатинин в моче	└┴┴┘ └┘ Ммоль/л	B12

РАСШИРЕННЫЙ МОДУЛЬ: Триглицериды и холестерин липопротеидов высокой плотности

28	Триглицериды	ммоль/л └─┴─┘.└─┴─┘	B13
29	Холестерин ЛПВП	ммоль/л └─┘.└─┴─┘	B14
